# Epigenetic signatures of smoking associate with cognitive function, brain structure, and mental and physical health outcomes in the Lothian Birth Cohort 1936

**DOI:** 10.1038/s41398-019-0576-5

**Published:** 2019-10-07

**Authors:** Janie Corley, Simon R. Cox, Sarah E. Harris, Maria Valdéz Hernandez, Susana Muñoz Maniega, Mark E. Bastin, Joanna M. Wardlaw, John M. Starr, Riccardo E. Marioni, Ian J. Deary

**Affiliations:** 1 Centre for Cognitive Ageing and Cognitive Epidemiology, Department of Psychology, University of Edinburgh, Edinburgh, EH8 9JZ UK; 2 Brain Research Imaging Centre, Edinburgh Imaging, University of Edinburgh, Edinburgh, UK; 3 Centre for Clinical Brain Sciences, University of Edinburgh, Edinburgh, EH16 4SB UK; 4 Centre for Genomic and Experimental Medicine, Institute of Genetics and Molecular Medicine, University of Edinburgh, Edinburgh, EH4 2XU UK; 5 Royal Victoria Building, Western General Hospital, Porterfield Road, Edinburgh, UK

**Keywords:** Clinical genetics, Predictive markers, Clinical genetics

## Abstract

Recent advances in genome-wide DNA methylation (DNAm) profiling for smoking behaviour have given rise to a new, molecular biomarker of smoking exposure. It is unclear whether a smoking-associated DNAm (epigenetic) score has predictive value for ageing-related health outcomes which is independent of contributions from self-reported (phenotypic) smoking measures. Blood DNA methylation levels were measured in 895 adults aged 70 years in the Lothian Birth Cohort 1936 (LBC1936) study using the Illumina 450K assay. A DNA methylation score based on 230 CpGs was used as a proxy for smoking exposure. Associations between smoking variables and health outcomes at age 70 were modelled using general linear modelling (ANCOVA) and logistic regression. Additional analyses of smoking with brain MRI measures at age 73 (*n* = 532) were performed. Smoking-DNAm scores were positively associated with self-reported smoking status (*P* < 0.001, eta-squared *ɳ*^2^ = 0.63) and smoking pack years (*r* = 0.69, *P* < 0.001). Higher smoking DNAm scores were associated with variables related to poorer cognitive function, structural brain integrity, physical health, and psychosocial health. Compared with phenotypic smoking, the methylation marker provided stronger associations with all of the cognitive function scores, especially visuospatial ability (*P* < 0.001, partial eta-squared *ɳ*p^2^ = 0.022) and processing speed (*P* < 0.001, *ɳ*p^2^ = 0.030); inflammatory markers (all *P* < 0.001, ranges from *ɳ*p^2^ = 0.021 to 0.030); dietary patterns (healthy diet (*P* < 0.001, *ɳ*p^2^ = 0.052) and traditional diet (*P* *<* 0.001, *ɳ*p^2^ = 0.032); stroke (*P* = 0.006, OR 1.48, 95% CI 1.12, 1.96); mortality (*P* < 0.001, OR 1.59, 95% CI 1.42, 1.79), and at age 73; with MRI volumetric measures (all *P* < 0.001, ranges from *ɳ*p^2^ = 0.030 to 0.052). Additionally, education was the most important life-course predictor of lifetime smoking tested. Our results suggest that a smoking-associated methylation biomarker typically explains a greater proportion of the variance in some smoking-related morbidities in older adults, than phenotypic measures of smoking exposure, with some of the accounted-for variance being independent of phenotypic smoking status.

## Introduction

Smoking is an exposure with broad and well-characterised adverse health effects. Smoking-associated death and disability remains a major global public health problem^1,2^. Understanding the mechanisms by which smoking predisposes individuals to chronic disease is crucial for the provision of therapeutic targets^3,4^, yet they are not well understood. Differential DNA methylation (DNAm) has been proposed as one possible partial explanation, which also could mean that these changes could act as a biomarker of smoking exposure. The possible attraction of a DNAm-based marker for smoking exposure lies in part with the limitations of other ways of quantifying smoking exposure. Analyses are usually dependent on self-report data, such as smoking status and pack years, which are prone to underestimation and reporting biases^5^. Cotinine, a metabolite of nicotine, is a widely used biomarker, but due to a half-life of around 15–20 h, it reflects only short-term exposure to smoke^6^.

Whether there is a direct dose-dependent association between smoking exposure and disease is debatable, as some studies have shown a non-linear relation with diseases such as coronary heart disease^3,7^ and cancer^8^. Although these studies show a trend for more cardiovascular events and cancer in active smokers, they have failed to find a significant dose-dependent correlation between risk, and the number of cigarettes smoked or the pack-years of exposure. This suggests that phenotypic measures of smoking are unable to capture the relevant smoking-related variance (the cumulative ‘hit’ from smoking, in lay terms) that relates to disease risk. These limitations underline the need for an objective measure of smoking exposure for precise classification in epidemiological studies. A better biomarker of smoking could also increase the effectiveness of interventions.

Smoking contributes to disease development and progression through genetic and epigenetic mechanisms^9^. DNAm is an epigenetic modification of the DNA molecule without altering the DNA sequence itself^10^. Epigenetic modifications are increasingly recognised as key mechanisms involved in response to environmental stimuli^11^, such as smoking, and in smoking-induced disease onset^12–17^. Smoking is robustly associated with highly specific DNAm changes at specific loci across the genome (occurring mainly at CpG—cytosine-phosphate-guanine—sites), which not only clearly distinguish between current and never smokers, but may also reflect the cumulative amount smoked, and time since quitting in former smokers^4,15,17–21^. In the majority of loci, smoking induces hypomethylation (loss of methylation)^15,22–24^. Recent evidence suggests that smoking-related DNAm changes occur after prolonged exposure to smoke (5–9 years for heavy smokers and 15–19 years for lighter smokers) and that these dose-dependent changes are reversible following cessation^19^.

Advances in epigenome-wide profiling of DNAm patterns associated with smoking have given rise to a new molecular biomarker or ‘epigenetic signature’ (epigenetic patterns detected in blood) of lifetime smoking exposure, with the potential to improve the prediction of smoking-related risks^13,15,25–31^. Differentially methylated loci with respect to smoking are related to clinical outcomes. Robust associations have been demonstrated between smoking-methylation signatures and major diseases including asthma^32^, COPD^33^, and lung cancer^34^, and markers of physical health, including lung function and periodontal disease^28^. Decreased methylation levels in the aryl hydrocarbon receptor repressor (AHRR) gene was found in the lung tissue of current smokers compared with non-smokers^35^. Smoking associated DNAm has also been shown to predict mortality across several studies, including a Scottish sample^36^, in coronary heart disease patients^37^, and in the ESTHER study in which a smoking-related DNAm score based on two CpGs (cg05575921 and cg06126421) showed strong associations with all-cause, cardiovascular, and cancer mortality^38^.

One previous study examined the relationships between self-reported smoking, serum cotinine and smoking-associated DNAm, and found that the smoking measures were correlated, and that the methylation marker was superior in measuring long-term smoking exposure based on its ability to discriminate between former smokers and never smokers with high accuracy^38^. Although several studies have examined smoking-related DNAm in relation to a specific outcome such as mortality^22^, see refs. ^25,30,31^, comparisons of the predictive value of epigenetic versus phenotypic smoking measures has never been performed simultaneously in the same sample, and for a range of health outcomes.

Here, we extend previous work by examining associations between smoking-associated changes in DNA methylation (smoking-DNAm scores), phenotypic smoking measures (current smoking status and pack years of smoking), and a comprehensive range of smoking-related health outcomes. We, (1) determine the proportion of variance the epigenetic and phenotypic predictors explain in their outcomes, (2) examine whether the smoking methylation marker accounts for variance in these outcome variables independently of the standard phenotypic smoking measures, and, (3) examine the life-course predictors of epigenetic and phenotypic smoking. The sample is a narrow-age cohort of older adults aged ~70 years at baseline, for whom there are extensive phenotypic data.

## Materials and methods

### Participants

Participants were from the Lothian Birth Cohort 1936 (LBC1936), a group of relatively heathy community-dwelling subjects in their seventies, enrolled in a longitudinal study of cognitive and brain ageing conducted in Scotland^39–41^. Most participants had previously taken part in the Scottish Mental Survey of 1947 (SMS1947^42^) at about age 11 years (from which we derived an age 11 IQ score), and subsequently traced and recruited to the study almost 60 years later, at approximately 70 years of age. Briefly, individuals born in 1936, who were living in the Lothian area of Scotland, were contacted by Lothian Health Board on behalf of the investigators and invited to take part in the study. In total, 1091 men and women were recruited at Wave 1 (2004–2007, age ∼70 years, *n* = 1091) with further follow-up waves at ages 73 (*n* = 866), 76 (*n* = 697), 79 (*n* = 550) and 82 (ongoing). Extensive phenotypic data have been collected, including blood biomarkers, cognitive testing, neuroimaging, and psychosocial, lifestyle, genetic, and health measures. All participants provided written informed consent before testing. The LBC1936 study was approved by the Multi-Centre Research Ethics Committee for Scotland (MREC/01/0/56) and the Lothian Research Ethics Committee (LREC/2003/2/29 for Wave 1 and 07/MRE00/58 for Waves 2–5).

Most of data for the present study come from Wave 1 (age 70). Structural brain imaging was undertaken three years later for 700 participants at Wave 2 (age 73). Here, a total of 895 individuals had smoking-DNAm data at age 70, and of the 895, 532 had MRI data at age 73. Following quality control which removed instances in which aberrant surfaces or segmentation errors were removed, additional analyses of cortical thickness were run for 521 participants.

### Epigenetic DNAm data

Blood samples were obtained at the time of Wave 1 baseline (age 70, *n* = 1091) assessment by trained research nurses using standard procedures, at the Wellcome Trust Clinical Research Facility Genetics Core at the Western General Hospital, Edinburgh. Of the 1091 LBC1936 participants, 1005 who had previously passed GWAS quality control were selected for methylation typing. Of these, 920 passed DNAm quality control. Due to missingness of measured cell counts (*n* = 14) and missing phenotype data (*n* = 11), this number dropped to the 895 that were included in the current analyses. DNAm typing was measured at 485,512 CpG sites using the Illumina Human Methylation450 Bead Chip (Illumina Inc., San Diego, CA). Full details of sample preparation and methylation typing have been reported previously^19,43^. Briefly, after background correction, probes were removed if they were poorly detected (*P* > 0.01) in >5% of samples or of low quality (via manual inspection). Samples were removed if they had a low call rate (*P* < 0.01 for <95% of probes), a poor match between genotype and SNP control probes, or incorrect DNAm-predicted sex.

A LASSO regression was performed to predict pack years of smoking on 3444 participants (73% current smokers, 27% never smokers) from the Generation Scotland study^19^. DNAm was assessed using the Illumina EPIC array in Generation Scotland although the data were subset to only consider CpG sites that were also present on the 450k array. Prior to the LASSO regression, the pack years phenotype was regressed on age, sex, and 10 genetic principal components. The optimal predictor utilised information from 233 CpG sites, 230 of which were available for analysis in the Lothian Birth Cohort 1936. Using the 230 CpG weights derived in McCartney et al., smoking epigenetic scores (trained to predict pack years of smoking) were created^19^. As pack years are positively coded, a higher methylation score indicates more smoking.

### Phenotypic data

#### Smoking

Self-report smoking status (never smoker, former smoker, current smoker) and smoking behaviour (age at starting, age at quitting, average number of cigarettes smoked per day) were ascertained at age 70 during a baseline interview. Pack years were calculated as the average number of cigarettes per day times years as a smoker, divided by 20, with zero assigned to never smokers. Pack years expresses lifelong exposure to cigarettes. Cotinine data were not available in the LBC1936.

#### Sociodemographic

Sociodemographic measures were education (number of years of formal full-time education), deprivation score at age 11 (derived from a combination of number of people sharing a room, inside or outside toilet, and number of people sharing the toilet), and adult occupational social class (highest status occupation classified as I-professional, to V-unskilled)^44^.

#### Cognitive function

Cognitive ability from childhood (age 11 IQ) was derived from scores on the Moray House Test no.12 (MHT), a validated test of general intelligence, obtained for the SMS1947^45^. MHT scores were corrected for age in days at time of testing and converted to standard IQ type scores, where mean = 100 and SD = 15. This is a general mental test comprising 71 items, mostly verbal reasoning, but also some numerical, spatial, and other items. Cognitive function measures at age 70 were age 70 IQ (the same test taken at age 11), the Mini-Mental State Examination^46^, and four latent scores representing: Visuospatial ability; Processing Speed; Memory; and Crystallised abilities. Visuospatial ability consisted of two subtests from the Wechsler Adult Intelligence Scale, 3rd UK Edition (WAIS-IIIUK^47^): Matrix Reasoning and Block Design. It also included the Spatial Span (Forward and Backward) subtest from the Wechsler Memory Scale, 3rd UK Edition (WMS-IIIUK^48^). Processing Speed was measured using two tests from the WAIS-IIIUK (Symbol Search and Digit-Symbol Substitution), Four-Choice Reaction time^49^, and Inspection Time (a computer-based task where participants must discriminate between two figures flashed on a computer screen for a variety of durations from 200 ms to 6 ms, then immediately backward-masked. There were 150 Inspection Time trials (10 at each of 15 durations), and the measure we used was the total number of correct responses^50^. Memory was measured using two subtests from the WMS-IIIUK (Verbal Paired Associates and Logical Memory), and the Digit Span Backward subtest of the WAIS-IIIUK. Crystallised Ability was measured by two tests that involved the participant reading aloud a list of irregular words: the National Adult Reading Test (NART^51^), and the Wechsler Test of Adult Reading (WTAR^52^). We also included a test of phonemic verbal fluency, using the letters C, F, and L^53^.

#### MRI measures

Brain structural MRI was first performed 3-years after baseline (when participants were ~age 73 years. Brain MRI acquisition and processing has been reported previously^54^. Briefly, a 1.5 T GE Signa HDx clinical scanner (General Electric, Milwaukee, WI, USA) was used to collect structural T1-, T2-, T2*-, and fluid attenuated inversion recovery-weighted images. Total brain volume (TBV), grey matter volume (GMV), white matter hyperintensity volume (WMHV), and normal-appearing white matter volume (NAWMV), are used in the present study. Measures were adjusted for intracranial volume (ICV) to control for head size. In addition, regional brain cortical thickness data were measured using FreeSurfer v5.1. Cortical thickness denotes the closest distance from the brain’s grey-white matter boundary to the grey-CSF boundary at each of 327,684 vertices. The sample lag between baseline assessment and MRI assessment was relatively small. That the phenotypic and epigenetic smoking variables were measured at the same time is more important for the purposes of comparison, i.e. the increased noise in the signal introduced by sampling lag is likely to be constant, and therefore unlikely to affect the relative differences in their magnitude.

#### Physical function

Physical function measures were: lung function (forced expiratory volume in one second FEV1) based on the highest score from three tests using a Micro Medical Spriometer); grip strength (based on the highest reading from the right hand using a North Coast Hydraulic Hand Dynamometer (JAMAR); walking speed (time in seconds to walk six metres at quickest pace); and body mass index (BMI) derived from height and weight (kg/m^2^). All measures were taken at time of assessment by trained nurses.

#### Biomarkers

Whole blood samples were drawn from participants on the day of assessment at the Western General Hospital, Edinburgh. Blood biomarkers used in the current study include: cholesterol (total cholesterol, HDL cholesterol, triglycerides, cholesterol ratio); inflammation (C-reactive protein (CRP), fibrinogen), and glycaemic status (glycated haemoglobin (HbA1c)). Serum cholesterol (mmol/L) was measured via non-fasting blood and analysed within 24 h in serum stored at 4 °C using an enzymatic Quinoneimine dye method measuring at 500 nm. The CRP (mg/L) assay was performed using a dry-slide immuno-rate method on OrthoFusion 5.1 F.S analysers (Ortho Clinical Diagnostics). The fibrinogen (g/L) assay was performed using an automated Clauss assay (TOPS coagulometer; Instrumentation Laboratory).

#### Psychosocial

Psychosocial measures were the HADS (Hospital Anxiety and Depression Score; anxiety and depression subscales, and total score^55^), and the WHOQoL (World Health Organisation Quality of Life physical, psychological, social relationships and environment subscales^56^).

#### Health behaviours

Health behaviour data, including alcohol intake (units/week) and dietary intake (dietary pattern scores), were derived from responses to a food frequency questionnaire (FFQ^57^). Dietary pattern scores were obtained previously via principal components analysis of all FFQ items (see ref. ^58^), and include: Mediterranean-style diet; Health-aware diet; Traditional diet; Sweet-foods diet.

#### Medical history

Binary variables relating to self-reported disease history include: cardiovascular disease (CVD); hypertension; diabetes; hypercholesterolaemia; and stroke. Deaths during follow-up (between 2004 and 2018) were identified by record linkage and coded as yes/no.

### Statistical analyses

The majority of the data used in the current study were collected at baseline (age ~70 years). MRI was performed age 73. Three-year stability in imaging markers in this cohort has been previously ascertained^59^. General linear modelling (ANCOVA) was used to investigate the associations between smoking measures (smoking-DNAm and phenotypic) and continuous outcome variables. An additive model included both smoking status and smoking-DNAm together. Logistic regression was used to investigate the associations between smoking and binary health variables (disease/no disease), namely: CVD; hypertension; high cholesterol; stroke; and, diabetes. The relationship between each smoking exposure and all-cause mortality was assessed using Cox proportional hazards models. We note that smoking-phenotypic associations with brain cortical thickness have been previously reported in this sample^60^ and are shown here for comparison with DNAm-smoking associations.

To examine the associations between life-course predictors of smoking in later life, we entered four life-course measures (age 11 deprivation score, age 11 IQ, education, and adult SES) into models, simultaneously, with smoking status (using logistic regression) and smoking-DNAm (using general linear models). We ran a series of path models within a structural equation modelling framework to assess the degree to which early-life factors (age 11 IQ and childhood deprivation) contributed to phenotypic and epigenetic smoking measures in later life, and whether their associations were mediated via years of education and adult SES-occupation. Specifically, we modelled the contributions of both childhood deprivation and childhood intelligence on education and adult SES, with the variables in a life-course order, and allowing a residual correlation between these two early-life factors. Contributions of all four predictors were modelled on smoking category and DNAm-smoking; that is, we fitted two models, one for each separate smoking outcome. The numerical values in the models’ results are standardised path coefficients, which may be treated like standardised partial beta weights.

All models were adjusted for age (exact age in days at time of testing) and sex. Height was included as an additional covariate in the models for FEV1, grip strength, and walking speed. All *p*-values were corrected for multiple comparisons using the false discovery rate (FDR) with an FDR corrected *p*-value ≤ 0.024 considered significant. We report partial-eta squared (*η*p^2^) effect sizes for ANCOVA models (to derive % variance explained) and *R*^2^, and odds ratios (OR) and 95% confidence intervals (95% CI) for logistic regression models. We report hazard ratios for associations between smoking and mortality using Cox proportional hazards regression. Most analyses were carried out using SPSS version 22. The brain cortical thickness linear regression analyses were conducted using the SurfStat toolbox (http://www.math.mcgill.ca/keith/surfstat) for Matrix Laboratory R2018a (The MathWorks Inc., Natick, MA). The path analyses were implemented using structural equation modelling which was conducted using ‘lavaan’ in R version 3.5.0^61^.

## Results

### Associations between smoking variables

Higher scores on the smoking-DNAm marker were strongly associated with higher self-reported smoking exposure. The Spearman correlation between DNAm scores and smoking pack years was *r* = 0.69 (*P* *<* 0.001). There was a significant association between DNAm and self-reported smoking status (F(2,892) = 764.03, *P* *<* 0.001, *ɳ*^2^ = 0.63). Thus, 63% of the variance in smoking-DNAm scores can be explained by phenotypic smoking status. The smoking DNAm values (mean ± sd) for never smokers was 3.08 ± 0.35, for former smokers was 3.71 ± 0.68, and for current smokers was 5.48 ± 0.74.

### Characteristics of the study sample

Table 1 shows the characteristics of the study sample (*n* = 895) by current smoking status. Of the total sample, 418 (47%) were self-reported never smokers, 375 (42%) were former smokers, and 102 (11%) were current smokers. The mean age of participants was 69.5 years (sd 0.8). Never smokers were more likely to be female and, compared with ever smokers, consumed less alcohol, had a higher childhood IQ and more education, and had fewer cases of CVD, and diabetes. Average pack years (cumulative smoking exposure) of current smokers was 45.0 and former smokers was 27.5. Current smokers had the highest prevalence of stroke and the lowest physical activity. Former smokers had the highest prevalence of CVD and diabetes, the highest BMI, alcohol consumption, and were most physically active. Over 14 years of follow-up, there were 224 (25%) deaths from 895 participants; 51% of current smokers at baseline had died, compared to 27% of former smokers and 17% of never smokers.

**Table 1:** Participant characteristics by smoking status

Characteristics	All participants	Smoking status			
		Never smokers	Former smokers	Current smokers	*P*
*n*	895	418	375	102	
Age, in years	69.5 ± 0.8	69.5 ± 0.9	69.6 ± 0.8	69.5 ± 0.7	0.92
Pack years of smoking^a^	6.5 ± 25.8	0	27.5 ± 29.0	45.0 ± 20.7	**<0.001**
DNAm smoking score^b^	3.6 ± 0.9	3.1 ± 0.3	3.7 ± 0.7	5.5 ± 0.7	**<0.001**
Education (years/full-time)	10.7 ± 1.1	10.9 ± 1.1	10.7 ± 1.1	10.5 ± 0.9	**<0.001**
Childhood (age 11) IQ^c^	99.8 ± 15.2	101.5 ± 15.2	98.6 ± 15.3	97.4 ± 14.8	**0.008**
Body mass index	7.8 ± 4.4	27.5 ± 4.1	28.5 ± 4.5	25.9 ± 4.6	**<0.001**
Alcohol consumption (units/week)	10.1 ± 9.2	8.3 ± 12.5	12.3 ± 14.4	9.2 ± 13.7	**<0.001**
Physical activity (days/month)	7.7 ± 8.1	7.8 ± 7.7	8.2 ± 8.6	5.2 ± 8.1	**0.009**
Sex (male)	453 (50.6%)	186 (44.5%)	220 (58.7%)	47 (46.1%)	**0.001**
CVD (yes)	218 (24.3%)	85 (20.3%)	105 (28.0%)	28 (27.5%)	**0.03**
Stroke (yes)	44 (4.9%)	17 (4.1%)	15 (4.0%)	12 (11.8%)	**0.003**
Diabetes (yes)	73 (8.2%)	24 (5.7%)	42 (11.2%)	7 (6.9%)	**0.02**
Hypertension (yes)	365 (40.8%)	169 (40.4%)	158 (42.1%)	38 (37.2%)	0.66
Hypercholesterolaemia (yes)	305 (34.1%)	127 (30.4%)	140 (37.3%)	38 (37.2%)	0.10
Deaths by 14 year-follow-up^d^	224 (25.0%)	72 (17.2%)	100 (26.7%)	52 (51.0%)	**<0.001**

We report mean ± standard deviation unless indicated as n (percent). *P*-values are derived from comparisons between categories of smoking status (never, former and current)

All results in bold-type are significant following FDR correction

*M* mean, *SD* standard deviation, *DNAm* DNA methylation, *CVD* cardiovascular disease

^a^Pack years of smoking was calculated for former and current smokers

^b^Higher methylation score = increased smoking exposure

^c^Childhood IQ was derived from a validated test of mental ability collected as part of the Scottish Mental Survey of 1947. Raw scores were corrected for age

in days at time of testing and converted to standard IQ type scores, where mean = 100 and SD = 15

^d^Number of deaths recorded between Nov 2004 and April 2018

### Smoking-DNAm and health outcomes

Table 2 shows the associations between phenotypic and epigenetic smoking and a range of health-related, cognitive, psychosocial, and lifestyle outcomes. Higher DNAm smoking scores were associated with significantly poorer outcomes in most of the domains tested (see Table 2 for full results). We summarise by reporting the % variance accounted for by smoking-DNAm. Higher DNAm was associated with lower cognitive function and poorer structural brain integrity: visuospatial ability (2.2%); processing speed (3.0%); crystallised abilities (0.8%); age 70 1Q (2.0%); lower TBV (4.0%); lower GMV (3.0%); lower normal appearing white matter volume (5.2%); and a higher volume of white matter hyperintensities (2.9%).

**Table 2:** Phenotypic smoking, epigenetic smoking, and phenotypic + epigenetic smoking, as predictors of health outcomes

	Phenotypic smoking						Epigenetic smoking			Additive model				
	Smoking status			Pack years			Smoking-DNAm			Smoking status		Smoking-DNAm		
	*P*	*ɳ*p^2^	*R* ^2^	*P*	*ɳ*p^2^	*R* ^2^	*P*	*ɳ*p^2^	*R* ^2^	*P*	*ɳ*p^2^	*P*	*ɳ*p^2^	*R* ^2^
Cognitive function														
Age 70 IQ	**0.001**	**0.015**	**0.018**	**<0.001**	**0.017**	**0.019**	**<0.001**	**0.020**	**0.022**	0.600	0.000	0.034	0.005	0.023
Visuospatial factor#	**<0.001**	**0.017**	**0.079**	**<0.001**	**0.015**	**0.074**	**<0.001**	**0.022**	**0.083**	0.776	0.001	0.033	0.005	0.083
Speed factor#	**<0.001**	**0.021**	**0.069**	**<0.001**	**0.020**	**0.067**	**<0.001**	**0.030**	**0.078**	0.788	0.001	**0.003**	**0.010**	**0.078**
Memory factor#	0.221	0.004	0.072	0.112	0.003	0.072	0.152	0.002	0.071	0.614	0.001	0.924	0.000	0.072
Crystallised factor#	0.398	0.002	0.050	**0.011**	**0.007**	**0.055**	**0.010**	**0.008**	**0.055**	0.450	0.002	**0.011**	**0.007**	**0.057**
Structural MRI measures														
TBV^a^	**0.002**	**0.024**	**0.110**	**<0.001**	**0.024**	**0.111**	**<0.001**	**0.040**	**0.125**	0.686	0.001	**0.002**	**0.018**	**0.126**
GMV^a^	**0.005**	**0.020**	**0.038**	**0.012**	**0.012**	**0.032**	**<0.001**	**0.030**	**0.048**	0.256	0.005	**0.005**	**0.015**	**0.053**
WMHV^a^	**0.006**	**0.019**	**0.029**	**<0.001**	**0.042**	**0.034**	**<0.001**	**0.029**	**0.039**	0.511	0.003	**0.010**	**0.012**	**0.042**
NAWMV^a^	**<0.001**	**0.038**	**0.069**	**<0.001**	**0.025**	**0.073**	**<0.001**	**0.052**	**0.083**	0.179	0.007	**0.001**	**0.021**	**0.088**
Physical function														
Lung function (FEV1)^b^	**<0.001**	**0.113**	**0.528**	**<0.001**	**0.142**	**0.542**	**<0.001**	**0.133**	**0.538**	0.029	0.008	**<0.001**	**0.030**	**0.542**
Grip strength (right hand)^b^	0.558	0.001	0.637	0.355	0.001	0.638	0.257	0.001	0.638	0.883	0.000	0.120	0.003	0.638
6-m walk time (s)^b^	**0.007**	**0.011**	**0.071**	**<0.001**	**0.026**	**0.083**	**<0.001**	**0.014**	**0.073**	0.780	0.001	0.100	0.003	0.074
Body mass index (kg/m^2^)	**<0.001**	**0.032**	**0.040**	**0.003**	**0.010**	**0.017**	**0.011**	**0.007**	**0.015**	**<0.001**	**0.025**	0.710	0.007	0.040
Bloods and biomarkers														
Total cholesterol (mmol/l)	0.186	0.004	0.112	0.042	0.005	0.115	0.139	0.002	0.111	0.456	0.002	0.525	0.000	0.113
HDL cholesterol (mmol/L)	0.036	0.008	0.104	**0.001**	**0.015**	**0.110**	0.096	0.003	0.099	0.131	0.005	0.673	0.000	0.104
Triglycerides (mmol/L)	**<0.001**	**0.017**	**0.024**	**<0.001**	**0.033**	**0.040**	**<0.001**	**0.017**	**0.024**	0.292	0.003	0.125	0.003	0.027
C-reactive protein (mg/L)	**<0.001**	**0.017**	**0.033**	**<0.001**	**0.028**	**0.044**	**<0.001**	**0.021**	**0.036**	0.631	0.001	0.052	0.004	0.037
Fibrinogen (g/L)	**<0.001**	**0.023**	**0.028**	**<0.001**	**0.016**	**0.022**	**<0.001**	**0.030**	**0.036**	0.682	0.001	**0.006**	**0.009**	**0.037**
HbA1C mmol/mol	0.061	0.006	0.012	**0.001**	**0.012**	**0.017**	0.058	0.004	0.009	0.359	0.002	0.834	0.000	0.012
Psychosocial														
HADS anxiety subscale	0.425	0.002	0.046	0.044	0.005	0.046	0.548	0.000	0.044	0.425	0.002	0.723	0.000	0.046
HADS depression subscale	**0.006**	**0.012**	**0.015**	**<0.001**	**0.016**	**0.020**	0.480	0.001	0.009	0.063	0.006	0.615	0.000	0.016
WHOQoL physical	**<0.001**	**0.032**	**0.034**	**<0.001**	**0.044**	**0.046**	**<0.001**	**0.017**	**0.020**	**0.001**	**0.017**	0.240	0.002	0.036
WHOQoL psychological	**<0.001**	**0.021**	**0.026**	**<0.001**	**0.028**	**0.033**	0.029	0.006	0.011	**0.001**	**0.018**	0.138	0.003	0.029
WHOQoL social relationships	**<0.001**	**0.017**	**0.022**	**0.004**	**0.011**	**0.015**	0.036	0.006	0.010	**0.006**	**0.013**	0.327	0.001	0.023
WHOQoL environment	**<0.001**	**0.041**	**0.046**	**<0.001**	**0.050**	**0.054**	**<0.001**	**0.024**	**0.029**	**0.001**	**0.018**	0.904	0.000	0.046
Lifestyle														
Alcohol intake (units/day)	**0.005**	**0.012**	**0.092**	0.123	0.003	0.081	0.210	0.002	0.083	**0.007**	**0.011**	0.352	0.001	0.093
Physical activity (days/month)	**0.011**	**0.012**	**0.014**	0.103	0.003	0.006	0.206	0.002	0.004	**0.014**	**0.011**	0.304	0.001	0.015
Mediterranean diet pattern	**0.001**	**0.018**	**0.047**	**0.011**	**0.009**	**0.039**	**0.018**	**0.008**	**0.037**	0.530	0.001	0.941	0.000	0.047
Healthy diet pattern	**<0.001**	**0.038**	**0.153**	**<0.001**	**0.034**	**0.147**	**<0.001**	**0.052**	**0.165**	0.708	0.001	**0.001**	**0.015**	**0.165**
Traditional diet pattern	**0.002**	**0.017**	**0.059**	**0.001**	**0.016**	**0.059**	**<0.001**	**0.032**	**0.074**	0.845	0.000	**0.001**	**0.016**	**0.074**
Sweet foods diet pattern	0.026	0.010	0.011	**<0.001**	**0.017**	**0.018**	**0.023**	**0.007**	**0.008**	0.165	0.005	0.231	0.002	0.013

All models used ANCOVA and were adjusted for age (exact age in days at time of testing) and sex

All results in bold-type are significant following FDR correction

*DNAm* DNA methylation, *TBV* total brain volume, *GMV* grey matter volume, *WMHV* white matter hyperintensity volume, *NAWMV* normal-appearing white matter volume, *FEV1* forced expiratory volume in 1 s, *HDL* high-density lipoprotein, *HbA1c* haemoglobin A1c, *HADS* Hospital Anxiety and Depression Scale, *WHOQoL* World Health Organisation Quality of Life

^a^MRI performed at age 73, all MRI measures were adjusted for intracranial volume (ICV)

^b^Measures were additionally adjusted for height

**Fig. 1:**
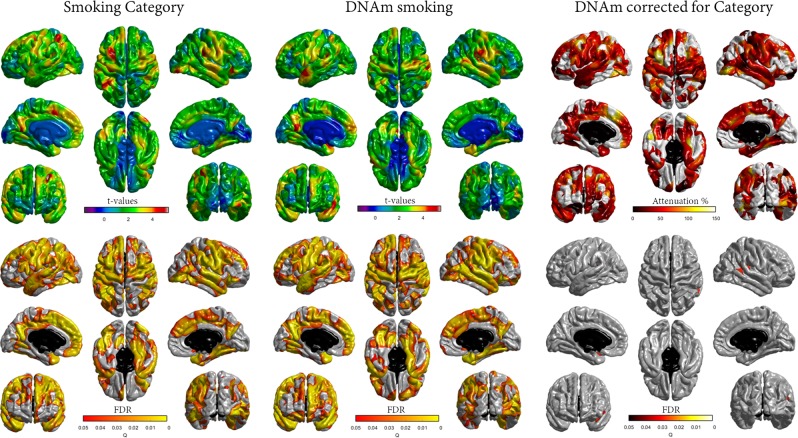
Associations between phenotypic and epigenetic smoking and cortical thickness. Figures denotes t-maps (top) and FDR q-values for age and sex corrected associations between smoking category (left) and DNAm-smoking (centre). Right hand panel shows the percentage attenuation (top) and FDR *q*-values (bottom) for the significant associations between DNAm-smoking and cortical thickness (shown in the centre panel) when also controlling for smoking category

Figure 1 shows associations between brain cortical thickness and phenotypic and epigenetic smoking. Higher smoking-DNAm was associated with a thinner brain cortex across a distributed network of regions including superior frontal and temporal cortices. The FDR-significant loci showed considerable overlap with areas which were also thinner in relation to smoking category.

Higher smoking-DNAm scores were associated with markers of poorer physical function and health: lower lung function FEV1 (1.3%); slower 6-m walk time (1.4%); higher BMI (0.7%); higher triglycerides (1.7%); higher CRP (2.1%) and fibrinogen (3.0%). DNAm was not associated with grip strength, total cholesterol, HDL cholesterol, or HbA1c. Higher smoking-DNAm scores were associated with lower psychosocial health (explaining 1.7% of the variance in WHOQoL-physical and 2.4% in WHOQoL-environment domain scores), and with poorer dietary patterns (explaining 0.8% of the variance in Mediterranean diet scores, 5.2% in ‘health-aware’ diet scores, and 3.2% in traditional diet scores). DNAm was also associated with lower quality of life on all of the domains tested; following correction for multiple testing, DNAm remained significantly associated with the WHOQoL Physical and Environment domains, accounting for 1.75 and 2.4% in these outcomes, respectively.

Table 3 shows that higher smoking-DNAm was also associated with a higher prevalence of stroke (OR 1.48, 95% CI = 1.12, 1.96, *P* *=* 0.006) and hypercholesterolaemia (OR 1.19, 95% CI = 1.02, 1.38, *P* *=* 0.024). Using Cox’s proportional hazards models, we observed a higher all-cause mortality risk (hazard ratio [HR] 1.59, 95% CI = 1.42, 1.79, *P* *<* 0.001). A positive association with mortality risk (HR 1.29, 95% CI = 1.05, 1.57, *P* *=* 0.013) has previously been shown in the LBC1936, over a slightly shorter (12 year) follow-up period^36^. Here, we showed that smoking-DNAm accounts for a proportion of the variance in stroke and mortality outcomes, which is independent of phenotypic smoking status.

**Table 3:** Phenotypic smoking, epigenetic smoking, and phenotypic + epigenetic smoking, as predictors of as predictors of disease outcomes and mortality

	Phenotypic smoking				Epigenetic smoking		Additive model			
	Smoking status^a^		Pack years		Smoking DNA-m		Smoking status		Smoking DNA-m	
	OR (95% CI)	*P*	OR (95% CI)	*P*	OR (95% CI)	*P*	OR (95% CI)	*P*	OR (95% CI)	*P*
CVD			1.18 (1.02, 1.36)	0.027	1.12 (0.96, 1.33)	0.143			1.03 (0.79, 1.35)	0.826
Ex-smoker	1.44 (1.03, 2.00)	0.032					1.41 (0.97, 2.05)	0.071		
Current smoker	1.48 (0.90, 2.44)	0.122					1.38 (0.61, 3.12)	0.440		
Hypertension			1.02 (0.89, 1.17)	0.756	1.05 (0.91, 1.21)	0.540			1.24 (0.97, 1.58)	0.087
Ex-smoker	1.06 (0.80, 1.42)	0.674					0.93 (0.67, 1.29)	0.672		
Current smoker	0.87 (0.56, 1.37)	0.552					0.52 (0.25, 1.10)	0.087		
Diabetes			1.20 (0.98, 1.47)	0.075	1.04 (0.80, 1.34)	0.791			0.93 (0.62, 1.39)	0.714
Ex-smoker	**1.96 (1.16, 3.32)**	**0.012**					**2.06 (1.15, 3.68)**	**0.015**		
Current smoker	1.20 (0.50, 2.88)	0.679					1.44 (0.39, 5.31)	0.582		
Hypercholesterolaemia			**1.19 (1.03, 1.36)**	**0.015**	**1.19 (1.02, 1.38)**	**0.024**			1.26 (0.99, 1.62)	0.064
Ex-smoker	1.32 (0.98, 1.78)	0.069					1.14 (0.81, 1.60)	0.449		
Current smoker	1.35 (0.86, 2.13)	0.193					0.77 (0.36, 1.63)	0.495		
Stroke			**1.38 (1.09, 1.74)**	**0.007**	**1.48 (1.12, 1.96)**	**0.006**			**1.15 (0.68, 1.93)**	**0.006**
Ex-smoker	0.96 (0.47, 1.96)	0.910					0.88 (0.40, 1.95)	0.749		
Current smoker	**3.20 (1.47, 6.96)**	**0.003**					2.31 (0.52, 10.22)	0.272		
Deaths^b^			**1.28 (1.16, 1.40)**	**<0.001**	**1.59 (1.42, 1.79)**	**<0.001**			**1.33 (1.08, 1.64)**	**0.007**
Ex-smoker	**1.58 (1.16, 2.14)**	**0.003**					1.30 (0.92, 1.83)	0.133		
Current smoker	**3.94 (2.75, 5.63)**	**<0.001**					1.41 (0.97, 2.05)	0.038		

All models used logistic regression and were adjusted for age (exact age in days at time of testing) and sex

All results in bold-type are significant following FDR correction

*DNAm* DNA methylation, *OR* odds ratios, *95% CI* 95% confidence intervals for the odds ratio

^a^Smoking status reference category = non-smoker

^b^Deaths recorded between November 2004 and April 2018, results analysed using Cox proportional hazards survival models

Compared with the phenotypic smoking measures epigenetic smoking accounted for a greater proportion of the variance for many of the significant smoking-health associations (see Table 2 for partial-eta squared values) including cognitive function, structural brain integrity, inflammatory markers, and dietary patterns.

### Phenotypic smoking and health outcomes

Smoking status and smoking pack years were significantly associated with most of the same outcome variables, and in the same direction, as epigenetic smoking (see Table 2). In addition to those outcomes, increased smoking exposure measured by the phenotypic smoking variables were also associated with poorer scores on some of the other psychosocial measures (HADS-depression score and WHOQOL-psychological and social relationship scores), a lower HDL cholesterol and a higher HbA1c level. Smoking status was associated with alcohol intake and physical activity but the results were not linear with smoking exposure (as previously reported in Table 1).

Compared with smoking-DNAm scores, the phenotypic smoking measures were generally stronger predictors of the psychosocial measures. We report higher effect sizes for phenotypic smoking and HADS-Depression score and all four quality of life subdomains of the WHOQOL). Both phenotypic smoking variables were associated with a higher prevalence of stroke (this association was significant for current smokers, (OR 3.20, 95% CI = 1.47, 6.96), *P* = 0.003), and for higher pack years (OR 1.38, 95% CI = 1.09, 1.74, *P* = 0.007). Higher pack years was associated with hypercholesterolaemia (OR 1.19, 95% CI = 1.03, 1.36, *P* = 0.015) (see Table 3), and past smoking, but not current smoking, was associated with diabetes (OR 1.96, 95% CI = 1.16, 3.32, *P* = 0.012). Associations of phenotypic smoking with CVD became non-significant following correction for multiple testing. Higher mortality risk over 14 years of follow-up was associated with higher pack years of smoking (HR 1.28, 95% CI = 1.16, 1.40, *P* < 0.001). Compared with never smokers, we found a higher mortality risk in former smokers (HR 1.58, 95% CI = 1.16, 2.14, *P* = 0.003), and current smokers (HR 3.94, 95% CI = 2.75, 5.63, *P* < 0.001).

### Additive model (smoking status + smoking-DNAm) and health outcomes

The additive model included both smoking status *and* epigenetic score simultaneously in order to examine whether the *R*^2^ was better than in the single predictor models. In none of the models were both predictors significantly associated with an outcome variable. For ten of the additive models, the smoking epigenetic score remained a significant predictor of health outcome measures even after smoking status was included. We report % variance accounted for in the outcome measure. These measures included processing speed (1.0%), crystallised ability (0.7%), structural brain MRI markers (range 1.2% to 2.1%), lung function FEV1 (3.0%), fibrinogen concentrations (0.9%), healthy diet pattern (1.5%) and traditional diet pattern (1.6%) (all Table 2), stroke (OR 1.15, 95% CI 0.68, 1.93, *P* = 0.006), and all-cause mortality risk (HR 1.33, 95% CI 1.08, 1.64, *P* = 0.007) (see Table 3).

Phenotypic smoking category remained a significant predictor, after DNAm smoking was included in the additive models, for BMI (2.5%), all of the WHOQoL measures (range 1.3–1.8%), alcohol intake (1.1%), physical activity (1.1%), and diabetes, for ex-smokers only, (OR 2.06, 95% CI 1.15, 3.68, *P* = 0.015).

**Fig. 2:**
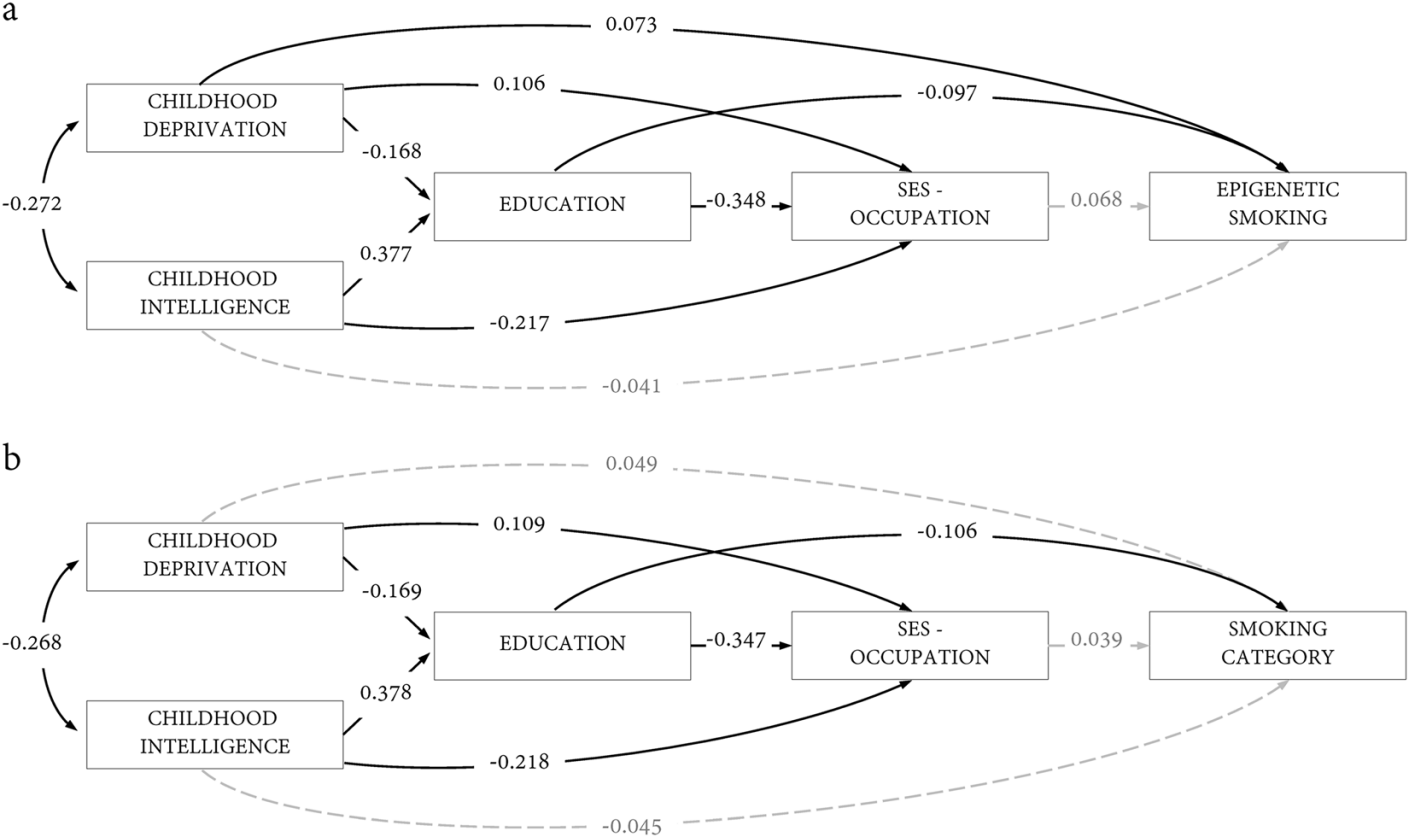
Path diagram for models of lifecourse predictors and smoking using structural equation modelling. Path coefficients are standardised

For vertex-wise brain cortical thickness, age and sex-corrected FDR significant associations between cortical thickness and smoking-DNAm were attenuated by an average of 33.3% when further corrected for phenotypic smoking category (Fig. 2). This also substantially reduced the spatial extent of FDR-corrected vertices, which were limited to only small clusters in left superior temporal and right supramarginal gyri.

### Life-course predictors of smoking

Table 4 shows the associations between life-course predictors and smoking using GLM (for epigenetic-smoking DNAm) and logistic regression (for phenotypic-smoking status). We entered life-course variables (age 11 deprivation, age 11 IQ, education, adult SES, plus age and sex) simultaneously into models in order to determine each predictor’s association with smoking pack years and smoking-DNAm. Education was the only significant predictor of smoking behaviour, accounting for 0.7% of the variance in smoking-DNAm, and for ex-smoking (OR 0.82, 95% CI 0.70, 0.96, *P* = 0.015), with the exception of adult SES, which was significant only for current smoking (OR 1.45, 95% CI 1.06, 1.99, *P* = 0.022).

**Table 4:** Life-course predictors of smoking status and smoking-DNAm

	Phenotypic smoking				Epigenetic smoking		
	Smoking status (former)^a^		Smoking status (current)^a^		Smoking-DNAm		
	OR (95% CI)	*P*	OR (95% CI)	*P*	95% CI	*P*	*η*p^2^
Deprivation score age 11	1.051 (0.985, 1.122)	0.130	1.029 (0.936, 0.113)	0.559	(0.001, 0.055)	0.041	0.005
IQ score at age 11	0.993 (0.981, 1.004)	0.202	0.995 (0.978, 1.012)	0.574	(−0.007, 0.003)	0.444	0.001
Education	**0.818 (0.695, 0.962)**	**0.015**	0.827 (0.632, 1.081)	0.165	**(−0.152, −0.016)**	**0.015**	**0.007**
Adult SES	0.837 (0.689, 1.017)	0.074	**1.450 (1.056, 1.992)**	**0.022**	(−0.008, 0.155)	0.078	0.004

Models used either logistic regression (phenotypic smoking) or linear regression (epigenetic smoking) and were adjusted for age (exact age in days at time of testing) and sex

All results in bold-type are significant following FDR correction

*DNAm* DNA methylation

^a^Smoking status reference category = non-smoker

Finally, path models were fit, using SEM, for each of the smoking outcome measures in order to examine the strength of associations between the life-course predictors and lifetime smoking behaviour, and to test whether any early-life associations were mediated via education and adult occupational status. The path diagrams are presented in Fig. 2. The standardised path coefficients show that smoking was directly and inversely associated with education (−0.106 for smoking category, and −0.097 for smoking-DNAm). We note that the total effect for education includes partial mediation via adult SES. We also note that education is moderately predicted by childhood intelligence (0.38). Therefore, the variables that contributed most to smoking behaviour were early-life measures rather than adult social position.

## Discussion

Using genome-wide DNAm values from the Illumina 450 K platform, we created a DNm biomarker of smoking and examined its ability to predict multiple smoking-associated adverse health outcomes in a healthy ageing cohort, the LBC1936. We found that higher smoking-DNAm scores were cross-sectionally associated with poorer cognitive function, physical function, psychosocial health, blood biomarkers of health, diet, and with markers of structural brain health measured at follow-up. Our analyses also showed that epigenetic signatures of smoking were associated with stroke, hypercholesterolaemia, and with a higher mortality rate after 14 years. The novel findings in the current study are, firstly, the epigenetic biomarker of smoking explained a greater proportion of the variance in many smoking-related morbidities than phenotypic smoking. The largest effect sizes for the methylation marker were observed for measures of cognitive function, structural brain integrity, lung function (FEV1), systemic inflammation, and mortality. Secondly, by combining the methylation predictor and self-reported smoking predictor in an additive model, we demonstrated that some of the accounted-for variance was independent of phenotypic smoking status. These findings support the predictive utility of a smoking-associated DNAm score compared with more traditionally used markers of smoking exposure for assessing smoking-related health risks. To our knowledge, this is the first study to compare the predictive capabilities of smoking-DNAm scores and conventional phenotypic self-report measures of smoking exposure, over a wide range of health-related outcomes, and in the same sample.

Quantification of smoking behaviour in epidemiologic studies, for the purposes of assessing smoking-attributable risk, is typically derived from questionnaire-based metrics of current and past smoking. Pack years is the most commonly used measure of smoking intensity. However, self-reported smoking data are hampered by recall bias, and as a socially undesirable behaviour, they are subject to under-reporting^62^. These data likely result in an underestimation of true effects^25^. Methylation derived scores reflect the cumulative physiological effects of smoking, compared with cotinine—an already present serological marker of smoking—which exclusively measures short-term exposure. Research into the distribution of methylation changes by time since smoking cessation, found that for many CpGs, methylation levels reverted back to levels of never smokers, but for some CpGs, hypo- and hypermethylation were still present 30–40 years after quitting^63^. Given the age of LBC1936 participants, most of whom were in their eighth decade at time of testing, smoking DNAm is a more informative and sensitive biomarker of lifetime smoking. As such, this objective, blood-based biomarker is more desirable for accurate evaluation and stratification of smoking-related disease risk, and has the potential to validate self-reports of smoking behaviour. Of prime importance is that, even in cohorts which have not collected phenotypic data on smoking, a DNAm-based measure can be used as a proxy for smoking exposure.

### Smoking-DNAm and cognitive function

In the current study, which benefits from a comprehensive assessment of cognitive function, the methylation-based biomarker of smoking better predicted deficiencies in visuospatial function and processing speed than either phenotypic measure (self-reported smoking status or pack years). Smoking is a well-established risk factor for cognitive decline^64^ and our results suggest that this biomarker may improve the ability to capture the deleterious effects of smoking exposure across major ageing-related domains of cognitive function, and provide valuable clues to disease pathways. The authors are unaware of any previous research to examine the link between smoking-associated DNAm and risk of cognitive decline but note that the precise regulation of DNAm is essential for normal cognitive function^65,66^. DNAm changes have been linked with the pathophysiology of brain ageing, Alzheimer’s Disease and other types of dementia^67^.

### Smoking-DNAm and MRI markers of brain health

We also observed that the epigenetic smoking score was a better predictor of decreased structural brain integrity in older age than the phenotypic markers of smoking. The strength of the associations between DNAm and brain MRI indices—accounting for between 3 and 5% of the variance in structural deficits such as reduced white matter integrity and cortical thinning—were striking given the time lag in measurement between baseline and MRI assessment. Moreover, that the smoking DNAm predictor explained variance in brain health independently of phenotypic smoking (in the additive model) may suggest that the methylation signatures are capturing additional effects of smoking which have neurobiological consequences. Previous neuroimaging studies have demonstrated widespread structural brain abnormalities in cigarette smokers, including ventricular enlargement^68^, cortical thinning^60^ (using the present sample), white matter hyperintensities^69^, reduced GMV^70^, and atrophy^71^. In a large UK Biobank study, pack years of smoking was related to a range of brain MRI measures including higher WMH volume, lower global and regional GMV, poorer white matter microstructure and lower subcortical volumes^72^. These structural MRI measures have been linked to an increased risk for dementia^73^. Differential brain structural measures, such as we observed here with higher DNAm, including lower overall brain volume, smaller GMV, reduced white matter integrity, increased WMHV, and greater cortical thinning, could indicate an effect of chronic nicotine exposure on pathological brain changes^74^. However, smoking-associated DNAm accounted for around double the proportion of variance in some of these brain volume measures compared with pack years, suggesting that a dose-response effect of lifetime nicotine exposure is less likely to be a cause, and rather, that DNAm better captures the neurological impact of chronic smoking.

### Smoking-DNAm and health

A number of novel smoking-DNAm biomarkers have been identified in recent years, using epigenome-wide association studies, which have been shown to be highly predictive for smoking-related health outcomes such as cancer and mortality^15,27,30,31,75–77^. In addition to cognitive and brain health, we also observed that smoking DNAm was a strong predictor of inflammatory marker concentrations, hypercholesterolaemia, stroke, and with all-cause mortality after 14 years.

We found that the smoking epigenetic score explained 2–3% of the variation in circulating CRP and fibrinogen. Smoking has a systemic impact and induces the release of pro-inflammatory markers^78^. Smokers have higher levels of CRP and Interleukin-6 (IL-6), but less is known about how epigenetic variation such as DNAm, correlates with chronic inflammation concentrations. Ligthart et al.^79^ performed a meta-analysis of EWAS of CRP and reported strong correlations with 58 methylation sites, and other studies report similar findings with other serum cytokines including IL-6 and tumour necrosis factor (TNF)^80^. Smoking-associated DNAm changes have previously been found in genes involved in inflammatory networks^81^ but the link between the epigenetic impact of smoking and inflammatory marker concentrations has not yet been evaluated in population studies. Given the crucial functions that inflammation has in brain ageing, neurodegeneration, and disease, it is important to discern whether smoking is driving the inflammatory response or whether this association is confounded by chronic disease.

Cigarette smokers are three to four times more likely to have a stroke^82^. In the current study, all three smoking measures were associated with a history of stroke, and the risk of stroke among those still smoking into their seventies, was over three times that of lifelong non-smokers, and in those with higher smoking DNA-methylation values. In the LBC1936 sample, neither epigenetic smoking nor phenotypic smoking variables were significant predictors of cardiovascular disease, a well-established health effect of prolonged smoking exposure. This in contrast with many previous studies which report associations between differential DNAm with smoking exposure, and various coronary artery diseases^9,31,37,76^.

On average, smokers die 13–14 years earlier than do non-smokers^83^, and differential methylation associated with smoking has been suggested as a potential mechanism. Zhang et al. reported clear dose-response relationships between smoking-related DNAm and mortality^30^, and in a further study, developed a biomarker strongly associated with all-cause mortality, cardiovascular, and cancer mortality^31^. Our results strongly support an increased risk of early death with smoking-associated DNm (also reported by McCartney et al.^36^), and we showed that current smokers had a fourfold increase in risk of death compared with lifelong non-smokers. Over half the current smokers at baseline (age 70) had died by follow-up approximately 14 years later, compared with 27% of ex-smokers, and 17% of never smokers. Smoking is associated with other unhealthy behaviours^84–86^, and lifestyle factors are associated with poorer health and mortality^87^. Here, we also observed significantly poorer dietary habits in those with higher smoking-DNAm values. To a lesser extent, poorer psychosocial health was observed in those with higher DNAm values in terms of lower quality of life associated with one’s physical health and environment.

### Life-course predictors of smoking

Interestingly, the epigenetic signatures of smoking at age 70 were associated with factors from early life, such as childhood deprivation, childhood cognitive function, and educational level. Furthermore, we showed that the variance explained by these childhood factors in smoking behaviour, were independent of phenotypic smoking. Previous studies have suggested that environmental influences including adversity in childhood have been linked with stable DNAm differences that persist into adulthood^88–92^. Early-life exposures (including those associated with SES) during sensitive periods may be stored in cells through epigenetic modifications that can be sustained for decades^93,94^. It is plausible that the accumulation of environmental exposures across the lifespan, contributes to epigenetic change with age. An alternative explanation could be that early-life factors, including low childhood IQ/high deprivation, leads to increased smoking uptake. Smoking is a strongly social-patterned risk factor; it is more prevalent among those with lower incomes and its association with economic, occupational and educational levels is well documented^95^. In epidemiological studies, it is often unclear whether the observed associations are true associations between epigenetic smoking signatures and poor health, or whether the associations are influenced by the socioeconomic path of an individual across the life-course, i.e., the result of poorer people ageing faster than more affluent people due to the unhealthy environments to which they are exposed^96^.

It is important to understand the life-course influences on such an important health-related variable as smoking, and we rarely have so many relevant, well-measured variables in one sample, across most of the human life-course. The LBC1936 study is relatively rare in having a direct measure of IQ from youth, multiple childhood deprivation data, education, and adult SES, especially in combination with both self-reported smoking and epigenetic smoking data. As far as the authors are aware, there have been no studies to date which have examined both childhood and adult SES influences, on smoking DNAm patterns. The results of the current study suggest that individual differences in smoking behaviour in later life, are best explained by education level rather than other life-course predictors. This finding supports previous studies that suggest education is the SES indicator that shows the greatest disparity in smoking outcomes^97^. However, the causal pathways between education and smoking are complex and subject to confounding from social networks, risk preferences and other factors. In addition, the initiation of regular smoking generally occurs before the completion of education. On the other hand, support for educational gradients in smoking come from those who argue that more schooling leads to the acquisition of important skills and resources that impact health management^98^.

### Strengths and limitations

The key strengths of our study include the use of a wide (almost ‘pheWAS’) array of traits examined in relation to smoking in a large single, narrow-age cohort study with DNAm. Given the age of participants, the DNAm score for smoking was based on many years of exposure, and is likely to be a more sensitive marker than in younger cohorts. Future analyses of longitudinal changes in smoking-DNAm in the LBC1936 are possible given that the study is ongoing. In terms of limitations, we must consider the causative versus correlative role of DNAm with respect to its relationship with age. It may be that the common changes in age-related epigenetic mechanisms across individuals are important contributors to the ageing process, rather than a consequence. Cotinine data were not available for this cohort, and therefore we were unable to compare the predictive capability of smoking DNAm with another smoking biomarker, or to validate self-reports of non-smoking. However, a single measure of cotinine concentrations is insufficient to reclassify participants into smoking categories given that it measures recent smoking only (previous 15–20 h), and LBC1936 participants (many of whom did not smoke daily) who refrained from smoking on the day of assessment or the day before, would have misleading data. Finally, the cross-sectional nature of the majority of our analyses limits our ability to make causal inferences and to study the time course of smoking effects. To that end, future studies with longitudinal data would be desirable to extend these findings in the current sample, and in other datasets, may partially explain the variable susceptibility to the health effects of cigarette smoking.

## Conclusions

Our study supports the potential utility of a smoking DNAm score, derived from genome-wide data, as a biomarker of lifetime smoking exposure, and for contributing toward the prediction of important ageing-related health outcomes in later life. In particular, the smoking methylation biomarker better predicted poorer cognitive function and brain structural integrity, chronic inflammation, stroke and mortality in later life, compared with much-used phenotypic measures of smoking. It may help to identify novel health impacts, improve adjustment for smoking in research studies, and shed light on the molecular mechanisms by which smoking predisposes to chronic mental and physical disease, and less good brain and cognitive health. In terms of clinical impacts, a methylation marker holds promise for better risk prediction in precision medicine. A useful implication of the present study is that it suggests that one may obtain an indication of smoking exposure and its implications even in studies which have not collected smoking data.

## Data Availability

LBC1936 data are available on request from the Lothian Birth Cohort Study, Centre for Cognitive Ageing and Cognitive Epidemiology, University of Edinburgh. LBC1936 data are not publicly available due to them containing information that could compromise participant consent and confidentiality.
